# High-Quality Single Crystalline Sc_0.37_Al_0.63_N Thin Films Enabled by Precise Tuning of III/N Atomic Flux Ratio during Molecular Beam Epitaxy

**DOI:** 10.3390/nano14171459

**Published:** 2024-09-08

**Authors:** Yuhao Yin, Rong Liu, Haiyang Zhao, Shizhao Fan, Jianming Zhang, Shun Li, Qian Sun, Hui Yang

**Affiliations:** 1 Key Laboratory of Semiconductor Display Materials and Chips, Suzhou Institute of Nano-Tech and Nano-Bionics, Chinese Academy of Sciences, Suzhou 215123, China; 2 Institute of Quantum and Sustainable Technology (IQST), School of Chemistry and Chemical Engineering, Jiangsu University, Zhenjiang 212013, China

**Keywords:** AlScN, scandium nitride, phase separation, ferroelectric, molecular beam epitaxy

## Abstract

We attained wurtzite Sc*_x_*Al_1−*x*_N (0.16 ≤ *x* ≤ 0.37) thin films by varying the Sc and Al fluxes at a fixed active nitrogen flux during plasma-assisted molecular beam epitaxy. Atomic fluxes of Sc and Al sources via measured Sc percentage in as-grown Sc*_x_*Al_1−*x*_N thin films were derived as the feedback for precise determination of the Sc*_x_*Al_1−*x*_N growth diagram. We identified an optimal III/N atomic flux ratio of 0.78 for smooth Sc_0.18_Al_0.82_N thin films. Further increasing the III/N ratio led to phase separation under N-rich conditions, validated by the observation of high-Sc-content hillocks with energy-dispersive X-ray spectroscopy mapping. At the fixed III/N ratio of 0.78, we found that phase separation with high-Al-content hillocks occurs for *x* > 0.37, which is substantially lower than the thermodynamically dictated threshold Sc content of ~0.55 in wurtzite Sc*_x_*Al_1−*x*_N. We postulate that these wurtzite-phase purity degradation scenarios are correlated with adatom diffusion and the competitive incorporation process of Sc and Al. Therefore, the Sc*_x_*Al_1−*x*_N growth window is severely restricted by the adatom kinetics. We obtained single crystalline Sc_0.37_Al_0.63_N thin films with X-ray diffraction (002)/(102) ω rocking curve full-width at half-maximums of 2156 arcsec and 209 arcsec and surface roughness of 1.70 nm. Piezoelectric force microscopy probing of the Sc_0.37_Al_0.63_N epilayer validates unambiguous polarization flipping by 180°.

## 1. Introduction

Recently, the development of epitaxial Sc-alloyed wurtzite III-nitrides has attracted significant research attention due to the enhancement of the *c*-axis piezoelectric response and spontaneous polarization [1]. By substituting Al sites in the wurtzite AlN lattice with Sc (Sc*_x_*Al_1−*x*_N), the coefficient of piezoelectric modulus d_33_ could be enhanced by a factor of 4–5 at a Sc content of 0.43 [2,3]. The increase in d_33_ relative to AlN translates into stronger electromechanical coupling in film bulk acoustic resonators (FBARs) and therefore broader bandwidth [4]. As the Sc content increases in the AlN matrix, the transition from wurtzite lattice towards layered hexagonal lattice is characterized by an increasing internal parameter from 0.38 to 0.50 [5] and boosted spontaneous polarization (2× for *x* = 0.40) [6], enabling versatile polarization engineering in III-nitride optoelectronic heterostructures. In 2019, S. Fichtner et al. demonstrated ferroelectric polarization switching with *x* ≥ 0.27 [7]. The unprecedently high paraelectric transition temperature of Sc*_x_*Al_1−*x*_N (>600 °C) enables stable operation under harsh environments [8]. Potential ferroelectric memory applications call for a lower coercive field in order to reduce power consumption [9], which demands higher Sc content close to the boundary of wurtzite-phase purity. Therefore, increasing the Sc composition in wurtzite Sc*_x_*Al_1−*x*_N alloys would have a drastic impact on the sub-6GHz resonant filter and high-temperature ferroelectric memory applications.

To date, multiple research groups have demonstrated single crystalline Sc*_x_*Al_1−*x*_N/GaN heterostructures grown via plasma-assisted molecular beam epitaxy (PA-MBE) with an Sc content of *x* ~ 0.18 targeting high-frequency power electronic applications [10,11,12,13]. The empirical MBE growth window has been investigated based on the beam equivalent pressures (BEPs) of Sc and Al sources, and “N-rich” conditions, i.e., (Sc_BEP_ + Al_BEP_) < N_BEP_ (N_BEP_ is an equivalent flux derived from AlN growth), were selected for Sc*_x_*Al_1−*x*_N alloy growth [11]. To maintain sufficient Al and Sc adatom diffusion for single crystalline growth, the elevated growth temperature (T_sub_ = 600–900 °C) in PA-MBE has been correlated with the degradation of wurtzite-phase purity in high-Sc-content Sc*_x_*Al_1−*x*_N (*x* > 0.30) thin films [14]; whereas the effect of precisely tuning Al, Sc, and active N atomic fluxes on Sc*_x_*Al_1−*x*_N phase separation remains elusive. Traditional high-Sc-content Sc*_x_*Al_1−*x*_N (*x* > 0.30) were deposited via magnetron sputtering at reduced temperatures (~400 °C), which exhibited fiber-textured characteristics, i.e., hexagonal columns aligned along the *c*-axis and quasi-random in-plane crystalline orientation [15,16,17,18]. Even though the pseudo-sixfold symmetry of in-plane orientation has been observed while molybdenum or tungsten buffer layers were used [9], sputtered Sc*_x_*Al_1−*x*_N (*x* > 0.20) layers generally require sufficient thickness (~1 μm) in order to achieve an X-ray diffraction (XRD) (002) ω rocking curve full-width at half-maximum (FWHM) of ~1° [16], and “undesired crystallites” in sputtered Sc*_x_*Al_1−*x*_N (*x* > 0.20) have a detrimental effect on the electromechanical coupling coefficient [19]. However, scatter loss due to grain boundaries in polycrystalline thin films reduces the resonance quality factor; and meanwhile, the further upscaling of the resonant frequency demands the downscaling of the film thickness [17]. Next-generation ferroelectric memory arrays also demand ultra-thin high-Sc-content Sc*_x_*Al_1−*x*_N (<200 nm, *x* > 0.30) with single crystallinity to reduce power consumption and to balance the requirements of miniaturization and reliability. To our knowledge, the most representative high-Sc-content Sc*_x_*Al_1−*x*_N (*x* > 0.30) thin films were reported by M. Hardy et al., with XRD (002) ω rocking curve FWHMs of 1840 arcsec for *x* = 0.32 [14] and 3190 arcsec for *x* = 0.39 [20], respectively. It is critical to elucidate the MBE growth diagram of wurtzite Sc*_x_*Al_1−*x*_N alloys at such high Sc composition further and to improve the crystalline quality.

In this work, we have investigated the PA-MBE growth window of Sc*_x_*Al_1−*x*_N thin films on the basis of the precise determination of Sc, Al, and active N atomic fluxes. Two distinctive types of phase separation were identified: (1) at a fixed Sc-to-Al atomic flux ratio for *x* = 0.18, undesirable Sc-agglomerated hillocks occur when the oversupply of active N is less than 15%; i.e., III/N ratio > 0.85; and (2) at a fixed III/N ratio of 0.78 for high Sc content (*x* > 0.30), Al-agglomerated hillocks start to emerge in the Sc*_x_*Al_1−*x*_N thin film when the Sc/Al atomic flux ratio is over 60%; i.e., *x* > 0.37. Both phase separation scenarios provide novel insight to the growth diagram of Sc*_x_*Al_1−*x*_N alloys, validating that the thermodynamic requirements of (i) N-rich synthesis conditions and (ii) Sc content *x* < 0.55 for stable wurtzite Sc*_x_*Al_1−*x*_N are inadequate at a growth temperature of 680 °C (pyrometer). The kinetic growth regime of PA-MBE, particularly the adatom surface diffusion and incorporation processes, could fundamentally affect the wurtzite-phase purity of Sc*_x_*Al_1−*x*_N. With this updated knowledge, we attained 100 nm thick single crystalline Sc_0.37_Al_0.63_N thin films with XRD (002) and (102) ω rocking curve FWHMs of 2156 arcsec and 209 arcsec and a mean square surface roughness (RMS) of 1.70 nm. Polarization switching by 180° is validated by applying a bias of ±15 V under piezoelectric force microscopy (PFM), promising potential applications in ferroelectrics. Our work provides guidance for the PA-MBE growth of high-Sc-content Sc*_x_*Al_1−*x*_N thin films and paves the way towards novel applications for the strongly polarized wide-bandgap material.

## 2. Experimental Details

Unintentionally doped Sc*_x_*Al_1−*x*_N films were grown on 2-inch Si(111) substrates and on GaN on sapphire (GaN/sapphire) templates using an in-house customized PA-MBE system (Fermion Instruments Corp., Ltd., Nanjing, P.R. China). A type-C thermocouple placed at 3 mm in proximity to the backside of the template/substrate was used for T_sub_ control; and meanwhile, a pyrometer was installed to monitor the temperature of template/substrate surface. To keep the growth temperatures of Sc_x_Al_1−x_N on Si(111) substrates and on GaN/sapphire templates comparable, we used the pyrometer to monitor the surface temperatures of both types of substrates, and the thermocouple temperature was adjusted to ensure a pyrometer temperature of 680 °C before the growth of Sc_x_Al_1−x_N, while the discrepancy in thermocouple temperatures of Si(111) substrates and of GaN/sapphire templates reached 30 °C. Once the growth started, we kept the thermocouple temperature constant even though the pyrometer temperature generally varied by 5–20 °C during Sc_x_Al_1−x_N growth. Standard SUMO cells were equipped with a cold-lip pyrolytic boron nitride (PBN) crucible for high-purity Al (6N5) ingots and two hot-lip PBN crucibles for Sc (4N) and Ga (7N5), respectively. The Al cell was heated to 1050–1100 °C to provide a beam equivalent flux (BEP) of 2.0 × 10^−8^–5.0 × 10^−8^ Torr, the Sc cell was heated to 1150–1250 °C to provide a BEP of 5.0 × 10^−9^–3.0 × 10^−8^ Torr, and the Ga cell was heated to 950–1000 °C to provide a BEP of 2.0 × 10^−7^–5.0 × 10^−7^ Torr. A 13.56 MHz inductively coupled N_2_ plasma cell was used with a mass flow controller to tune high-purity N_2_ (6N) gas at 0.35 standard centimeters per minute (sccm) and with a radio-frequency impedance matching box to sustain a plasma power of 350 W. The atomic flux of active nitrogen was calculated using an AlN growth rate at 180 nm/h grown under metal-rich conditions. The MBE chamber was equipped with in situ reflection high-energy electron diffraction (RHEED, Dr. Glasser electron devices GmbH, Ulm, Germany).

Two types of substrates, Si(111) substrates and commercial 4 μm thick GaN/sapphire templates grown via metal organic vapor-phase epitaxy (MOCVD), were utilized for Sc*_x_*Al_1−*x*_N growth. Hoglund et al. reported the effect of the substrate on the achievable Sc composition of pure wurtzite phase [21,22], demonstrating correlation between strain energy and phase stability [23]. K. Yazawa et al. and V. Gund et al. reported that in-plane tensile film stress can effectively push the phase separation boundary of wurtzite Sc*_x_*Al_1−*x*_N towards higher Sc percentage [9,24]. Herein, we focus on the effect of III/N on Sc*_x_*Al_1−*x*_N film growth kinetics, while the proper choice of substrate for different Sc compositions enables a similar strain state during Sc*_x_*Al_1−*x*_N nucleation.

Samples C1–C4 with Sc content *x* = 0.18 were grown on Si(111) substrates using a 50 nm thick AlN buffer layer to mimic the compressive strain state of Sc*_x_*Al_1−*x*_N (*x* > 0.18) grown on GaN/sapphire templates. The Si(111) substrate was outgassed at 800 °C for 1 h in the preparation chamber, and after transfer to the growth position, it was baked at 900 °C for 30 min prior to growth. Samples C5–C9 with Sc content *x* varying from 0.16 to 0.39 were grown on GaN/sapphire using a 100 nm thick GaN buffer layer. The backsides of the GaN/sapphire templates were sputter-coated with 2 μm thick titanium to enhance heat absorption. The GaN/sapphire templates were cleaned in ultrasonic baths of acetone and isopropanol, rinsed with deionized water, and blown dry in dry N_2_ prior to their loading into the MBE loadlock. Subsequently, the templates were outgassed at 450 °C for 2 h in the preparation chamber and at 600 °C for 30 min in the growth chamber prior to growth.

Surface morphology of as-grown samples was characterized using a Bruker ICON atomic force microscope (AFM, Bruker Corp., Billerica, United States) in tapping mode and a Hitachi S4800 scanning electron microscope (SEM, Hitachi High-Tech Corp., Hitachinaka, Japan). Sample thickness was measured via cross-sectional SEM. The Sc content *x* was determined via two independent approaches: X-ray diffraction (XRD) and X-ray photoelectron spectroscopy (XPS). XRD 2θ-ω scans and ω rocking curve scans were performed on a PANalytical X’pert3 MRD high-resolution X-ray diffractometer equipped with Cu-K_α_ radiation (λ = 1.54056 Å at 40 kV and 45 mA), a Ge symmetric four-bounce monochromator, and a scintillator detector (Malvern Panalytical. Co., Malvern, UK). A Thermo Fisher ESCALAB QXi XPS system with an Al-K_α_ X-ray source (1486.6 eV) was used for chemical composition analysis (ThermoFisher Scientific, Waltham, MA, USA). Energy dispersive X-ray (EDX) spectroscopy was acquired using a FEI Nova Nano SEM 450 system equipped with an Octane Super EDX detector (AMETEK Inc., Berwyn, IL, USA). PFM measurements were conducted using a commercial atomic force microscope (Asylum Research MFP-3D, Oxford Instruments, Santa Barbara, CA, USA). The amplitude and phase patterns were mapped with a writing voltage of ±15 V, and the GaN buffer layer was grounded. An AC voltage with an amplitude of 600 mV and a contact resonance frequency of approximately 30 kHz was applied to the tip during PFM measurement.

## 3. Results and Discussion

### 3.1. Effect of III/N Atomic Flux Ratio on Sc_0.18_Al_0.82_N Growth

The thermodynamic preference of intermetallic Al*_x_*Sc*_y_* phases dictates N-rich growth conditions for Sc*_x_*Al_1−*x*_N [12,13]. Z. Engel et al. demonstrated that under a high nominal III/N ratio of ~1.5, catalytic cracking of N_2_ by accumulated metallic Sc effectively reduces III/N atomic ratio at the growth front [25]. This effect is largely suppressed under N-rich conditions. In previous studies on the MBE growth of Sc*_x_*Al_1−*x*_N films, the optimal III/N ratio values were scattered in a range of 0.50–0.85; they were determined by the sum of Sc and Al BEPs over the active nitrogen flux derived from the Al-rich growth of AlN [14,26,27]. However, depending upon the geometrical configuration of the MBE reactor and the responsivity of the flux monitor, the *normalization factor* between the true atomic flux and the nominal BEP could be radically different for Sc and Al sources. As such, it is necessary to calibrate the true atomic flux of Sc and Al, which can be derived from the film thickness and Sc composition of as-grown Sc*_x_*Al_1−*x*_N. Specifically, the *normalization factor* for the Sc source reaches 65.0% that of the Al source in our MBE chamber, whereas the *normalization factor* for the Sc source was calibrated to be 97.7% that of the Al source, as reported by K. Frei et al. [12]. Herein, we utilized the atomic flux ratio of III/N as a universal gauge to investigate MBE growth diagram.

**Figure 1:**
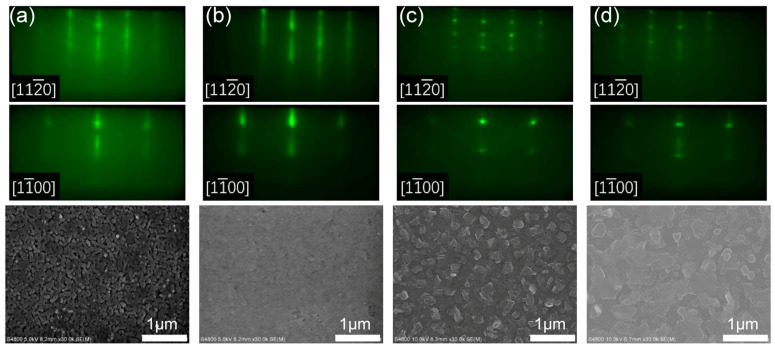
Reflection high-energy electron diffraction (RHEED) and planar-view scanning electron microscope (SEM) images of Sc_0.18_Al_0.82_N films (samples C1–C4) grown on Si (111) substrates with different III/N atomic flux ratios. RHEED patterns of Sc_0.18_Al_0.82_N epilayers along [$11\bar{2}$0] and [$1\bar{1}$00] during growth are shown. The III/N ratios are (**a**) 0.69 for sample C1, (**b**) 0.78 for sample C2, (**c**) 0.89 for sample C3, and (**d**) 1.12 for sample C4.

Our comparison of Sc_0.18_Al_0.82_N films grown under various III/N atomic flux ratios of 0.69–1.12 demonstrates that streaky RHEED patterns and a smooth surface can be achieved at an optimal flux ratio at 0.78, and that a further increase in III/N ratio, even within the N-rich regime, leads to spotty RHEED patterns and densely packed hillocks across the surface. At III/N = 0.69 (sample C1), the planar-view SEM in Figure 1a shows the incomplete coalescence of individual crystallites, resembling columnar microstructures with a lateral grain size of 100–300 nm. The corresponding RHEED patterns exhibit streaks and arcs, indicating in-plane angular twisting of grains [20]. By increasing the III/N ratio to 0.78 (sample C2), RHEED patterns in Figure 1b exhibit sharper streaks without any arcs. The significantly improved film smoothness implies enhanced adatom mobility, promoting the growth mode from 3D to 2D. At even higher III/N = 0.89 (sample C3), we observed densely packed hillocks of ~300 nm in diameter across the surface, while the RHEED patterns became spotty with emerging ring-like characteristics. Further increasing the III/N ratio to the metal-rich regime (sample C4) led to similar but slightly dimmer RHEED patterns; meanwhile, the surface hillocks evolved towards a coalesced terrace, exhibiting elongated hexagonal features. No Sc dendrites or intermetallic droplets can be observed in Figure 1c,d [12,25].

We conducted EDX scans to investigate elemental distribution of Al and Sc across the films. EDX spectra taken under accelerating voltages of 5 keV and 10 keV were shown in Figure S1 (Supplementary Material S1). The electron energy of 5 keV in the primary beam is slightly higher than the critical excitation energy of the K shell in Sc (4.492 keV). An overvoltage ratio of >2 is required for the efficient generation of Sc-K_α_ radiation (4.093 keV). As a result, the peak of Sc-K_α_ radiation (4.093 keV) is fairly weak, and the primary peak in the EDX spectrum of 5 keV is Sc-L_α_ radiation (0.395 keV), which is indistinguishable from the N-K_α_ radiation (0.392 keV) considering a spectral resolution of ~100 eV. The beam penetration depth within III-nitrides is approximately 200 nm for an accelerating voltage of 5 keV and 600 nm for 10 keV [28]. At 10 keV, the intensity of Sc-K_α_ radiation (4.093 keV) was enhanced by a factor of 2.5× that at 5 keV. Given the reduced electron beam interaction with the underlying AlN buffer layer, the intensity of Al-K_α_ radiation (1.486 keV) at 5 keV is 6.4× that at 10 keV. Therefore, we chose an accelerating voltage of 5 keV for elemental mapping in order to maximize the contrast due to the inhomogeneous distribution of Al; meanwhile, we conducted EDX line scans at 5 keV to boost the intensity of Sc-K_α_ radiation.

**Figure 2:**
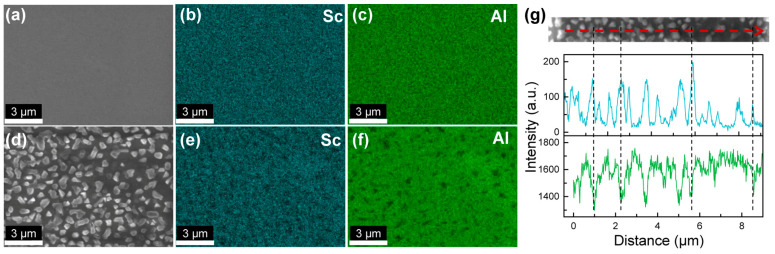
Energy-dispersive X-ray (EDX) spectroscopy analysis of sample C2 (III/N = 0.78) and sample C3 (III/N = 0.89): (**a**) SEM, (**b**) Sc, and (**c**) Al mappings of sample C2; (**d**) SEM, (**e**) Sc, and (**f**) Al mappings of sample C3; (**g**) EDX line scan of sample C3 along the dashed red line showing that the hillocks have higher Sc and lower Al percentages with respect to the background. The electron beam energy is 10 keV for all EDX mappings and the line scan of Al (green). The beam energy is 5 keV for the line scan of Sc (cyan) in (**g**).

EDX mappings in Figure 2 demonstrate that sample C2 grown at III/N = 0.78 has uniform Sc and Al distribution across the surface (see line scans in Figure S2 of Supplementary Materials), whereas the hillocks in sample C3 (III/N = 0.89) are characteristic of phase separation. Comparison between the SEM image in Figure 2d and the Al mapping in Figure 2f shows that the hillocks are Al-deficient microstructures with respect to the background. In Figure 2g, the EDX line scans further confirm that those Al-deficient hillocks have a higher Sc percentage compared to the surrounding regions. Considering the relatively low target Sc content *x* = 0.18, this type of phase separation could potentially be driven by surface kinetics [29]. III/N atomic flux ratio governs adatom mobility, and an N-stable surface has a high energy barrier for Sc and Al adatom diffusion [30,31]. We hypothesize that increasing III/N promotes higher Sc adatom mobility. The Sc adatoms, if not instantaneously incorporated into the metastable wurtzite lattice, could have a better chance to form the thermodynamically more-preferential rock-salt phase ScN, high-Sc-content Sc_1−*y*_Al*_y_*N alloys, or intermetallic-phase Al_3_Sc during its diffusion at the growth front [23]. Indeed, Figure S3 shows that the XRD (002) 2θ-ω scan of sample C3 exhibits a peak at ~38.0° due to metallic cubic-phase Al_3_Sc [25], which is absent in the scan of sample C2. Both ScN and Al_3_Sc exhibit higher a Sc percentage compared to the target Sc content of *x* = 0.18; therefore, we observed high-Sc-content hillocks via EDX.

### 3.2. Characterization of Sc Composition and Crystal Quality of Sc_x_Al_1−x_N Films

**Figure 3:**
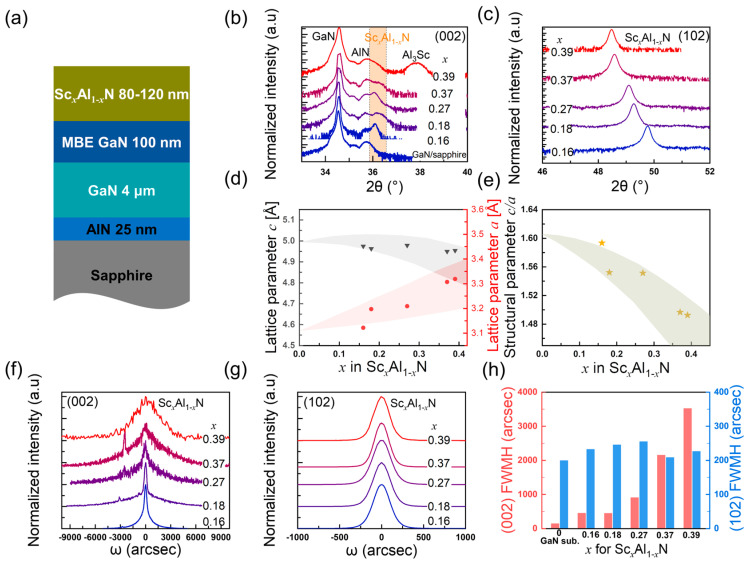
Structural characterization of Sc*_x_*Al_1−*x*_N thin films (samples C5–C9) grown on GaN/sapphire templates via X-ray diffraction (XRD): (**a**) structural schematics of the samples; (**b**) (002) and (**c**) (102) 2θ-ω scans of the samples (the (002) 2θ-ω scan of the GaN/sapphire template is included in (**b**) to show the peaks of GaN and AlN); (**d**) lattice constant *a* and *c* versus Sc content in wurtzite Sc*_x_*Al_1−*x*_N calculated assuming no residual strain; (**e**) calculated structural parameter *c*/*a* versus Sc content; (**f**) (002) and (**g**) (102) ω rocking curve scans of the samples; (**h**) full width at half-maximum (FWHM) of the ω rocking curves in (**f**,**g**). The calculated Sc content is used to label each sample in other images. The shadows in (**d**,**e**) indicate the ranges of the lattice parameters or structural parameters reported in the literature [6,32,33].

**Figure 4:**
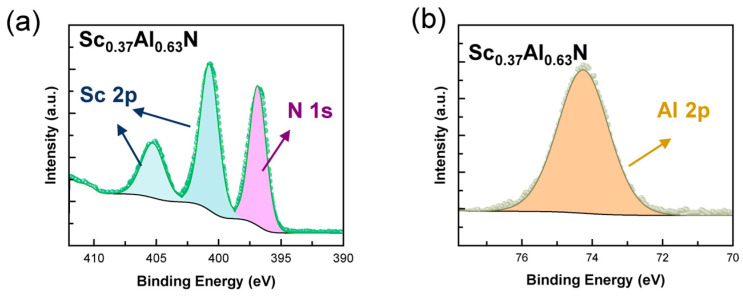
X-ray photoelectron energy spectroscopy (XPS) of the Sc_0.37_Al_0.63_N thin film grown on GaN/sapphire templates (sample C8). Zoomed-in XPS spectra show (**a**) Sc 2p_3/2_ peak at 400.9 eV, Sc 2p_1/2_ peak at 405.3 eV, and N 1s peak at 396.9 eV; and (**b**) Al 2p peak at 74.3 eV.

Structural characterization via XRD verified that the Sc composition of pure wurtzite Sc*_x_*Al_1−*x*_N films grown on GaN/sapphire templates can be tuned from 0.16 to 0.37, and further increasing the Sc-to-Al atomic flux ratio leads to phase separation. In Figure 3b, the (002) 2θ-ω scan peaks of Sc*_x_*Al_1−*x*_N are in the range of 35.9–36.5°, while the three peaks on the lower angle side are 34.5° from the GaN buffer layer, 35.7° from the sputtered 25 nm thick AlN nucleation layer with compressive strain, and 35.2° due to the satellite peak of AlN, respectively. For sample C9, the peak at 37.9° is likely from the metallic-phase Al_3_Sc [25]. We observed slight variation in peak positions of samples C5–C9, indicating a lattice constant *c* in the range of 4.95–4.98Å. Our observation is consistent with the literature reports that *c* gradually increases by <1% with Sc composition from *x* = 0 to ~0.25 and then decreases [6,32,33]. We conducted (102) 2θ-ω scans of Sc*_x_*Al_1−*x*_N with skew angles of 42.6–40.8°. The structural deviation of Sc*_x_*Al_1−*x*_N from GaN corresponds to a discrepancy of 0.6–2.4° in the angle between (002) and (102) planes, and as a result, no GaN (102) peak was observed in Figure 3c. The lattice constant *a* was derived and plotted with *c* in Figure 3d versus the Sc composition calculated assuming no residual strain in the Sc*_x_*Al_1−*x*_N films. The Sc content of sample C9 is approximately the same as sample C8, even if the target Sc content based on the Sc-to-Al atomic flux design is higher at *x* = 0.44. Our observation of Sc composition saturation in the wurtzite phase, together with the emergence of the intermetallic-Al_3_Sc phase in the XRD (002) 2θ-ω scan [Figure 3b] and dual spots in RHEED patterns, indicates that the achievable Sc content for pure-wurtzite Sc*_x_*Al_1−*x*_N by PA-MBE is *x* = 0.37 under a T_sub_ of 680 °C. We surveyed representative Sc*_x_*Al_1−*x*_N samples via XPS, as shown in Figure 4, for sample C8. We analyzed the elemental composition after removing surface contaminants and oxides via an argon ion gun. The peaks with binding energies of 74.3 eV, 396.9 eV, 400.9 eV, and 405.3 eV correspond to Al 2p, N 1s, Sc 2p_3/2_, and Sc 2p_1/2_, respectively. The Sc content *x* of Sc*_x_*Al_1−*x*_N was calculated from the peak areas of Sc 2p and Al 2p using the software Advantage (version 5.9931), which accounts for the sensitivity factors of each element. Subsequently, the ratio of the normalized peak areas of Sc 2p and Al 2p was used to determine the relative percentage of Sc in the Sc*_x_*Al_1−*x*_N film, yielding a value of 38% for sample C8. The Sc composition derived from XRD is consistent with the XPS quantification within an error of ±1% (abs.). Figure 3e shows that the structural parameter *c*/*a* versus Sc composition decreases from 1.59 to 1.50 as *x* increases from 0.16 to 0.37, in comparison to the value of 1.60 for AlN. Although spontaneous polarization presents in an ideal wurtzite structure with *c*/*a* = 1.63 [34], structural nonideality, symbolized by a reduced *c*/*a* value, has a significant contribution to spontaneous polarization [35]. Therefore, the structural parameter reduction might have great potential for polarization engineering.

**Figure 5:**
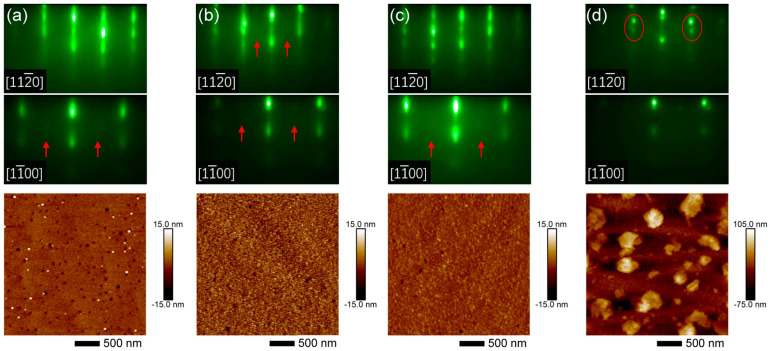
RHEED and atomic force microscopy (AFM) images of Sc*_x_*Al_1−*x*_N thin films (samples C6–C9) grown on GaN/sapphire templates with a fixed III/N atomic ratio of 0.78 and different Sc-to-Al atomic flux ratios. The Sc-to-Al atomic flux ratios are (**a**) 20% (sample C6, Sc_0.18_Al_0.82_N), (**b**) 40% (sample C7, Sc_0.27_Al_0.73_N), (**c**) 60% (sample C8, Sc_0.37_Al_0.63_N), and (**d**) 80% (sample C9, Sc_0.39_Al_0.61_N), respectively. High-order 2× streaks are indicated by red arrows, and dual spots in the red circles indicate reflection from the (111) and (002) planes of rock-salt-phase Sc*_x_*Al_1−*x*_N. The mean square roughness (RMS) of each 3 μm × 3 μm AFM image is (**a**) 1.50 nm, (**b**) 3.98 nm, (**c**) 1.70 nm, and (**d**) 24.50 nm.

Comparison of XRD ω rocking curve scans in Figure 3f,g reveals that the FWHMs of (002) scans have a marked degradation with increasing Sc composition, whereas the FWHMs of (102) scans are relatively constant in the range of 200–250 arcsec for various Sc compositions. At a low Sc content *x* < 0.20, the (002) FWHM of 450 arcsec increases by a factor of 3× with respect to that of the GaN. At *x* = 0.27, 0.37, and 0.39, (002) the FWHMs are 911 arcsec, 2156 arcsec, and 3524 arcsec, indicating a strong correlation between increasing screw/mixed dislocation density and decreasing lattice constant *c*. For reference, P. Wang et al. reported (002) FWHMs of 2200 arcsec for *x* = 0.30 and 2800 arcsec for *x* = 0.38 [11]; Hardy et al. reported XRD (002) FWHMs of 1840 arcsec for *x* = 0.32 [14] and 3190 arcsec for *x* = 0.39 [20]. Comparison of (002) and (102) FWHMs of our samples with those high-Sc-content Sc*_x_*Al_1−*x*_N epilayers in the literature can be found in Table S1 (Supplementary Material S3). Sc*_x_*Al_1−*x*_N grown under even higher Sc-to-Al atomic flux ratios is characterized by 3D nucleation islands and lateral coalescence, which is evident by the spotty RHEED patterns in Figure 5d. The *c*-axis tilt of neighboring crystallites generates arrays of geometrically necessary screw/mixed threading dislocations at low-angle coincidence grain boundaries [36]. The drastic increase in the (002) FWHM is a direct indicator of deteriorating random *c*-axis tilting, potentially due to inhomogeneous softening the wurtzite lattice with increasing Sc [37]. Consequently, a reduction of piezoelectricity is expected with *c*-axis tilting [38,39]. Surprisingly, negligible degradation has been observed in the (102) FWHMs, which implies superior epitaxial registry with the underlying GaN buffer layer. The ability to maintain in-plane crystalline orientation and to suppress fiber textures via MBE growth kinetics in comparison to physical sputtering techniques could be a distinctive advantage in device operation that relies on in-plane properties such as d_31_ [40].

### 3.3. High Sc-Content Sc_x_Al_1−x_N Films and Phase Separation

**Figure 6:**
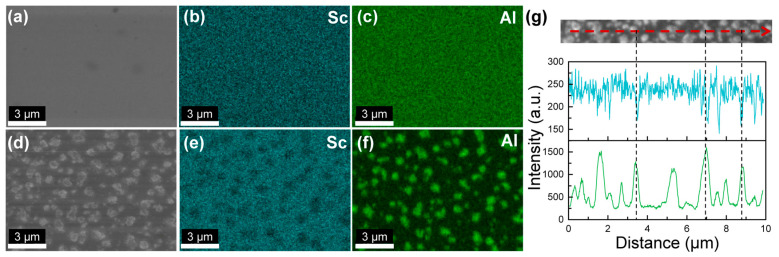
EDX spectroscopy analysis of sample C8 and sample C9: (**a**) SEM, (**b**) Sc, and (**c**) Al mappings of sample C8; (**d**) SEM, (**e**) Sc, and (**f**) Al mappings of sample C9; (**g**) EDX line scan of sample C9 along the dashed red line showing that the hillocks have lower Sc and higher Al percentages with respect to the background. The electron beam energy is 10 keV for all EDX mappings and the line scan of Al (green). The beam energy is 5 keV for the line scan of Sc (cyan) in (**g**).

Figure 5 demonstrates the evolution of the RHEED patterns and surface morphology of Sc*_x_*Al_1−*x*_N films grown on GaN/sapphire templates with the increasing Sc-to-Al atomic flux ratio. We observed spotty-modulated-streaky RHEED patterns with faint high-order 2× streaks (indicated by red arrows) for *x* = 0.18–0.37 (samples C6–C9), signifying the presence of surface-reconstruction during growth. No ring-like features were present in the RHEED patterns, consistent with the low XRD (102) ω rocking curve FWHMs in Figure 3h. Samples C6–C8 exhibit a relatively smooth surface, and the increase in Sc content has a subtle effect on the surface microstructure. For *x* = 0.18 (sample C6), the surface has a granular structure decorated with pits and hillocks of 30–70 nm in diameter [Figure 5a]. A pit density of the order of 10^8^ cm^−2^ was also observed from the GaN buffer layer, potentially related to impurities on the substrate surface [41] or screw/mixed dislocations. In Figure 5a, the density of pits approximates 1.4 × 10^9^ cm^−2^, higher than the screw/mixed dislocation density of 4.5 × 10^8^ cm^−2^ derived from XRD ω rocking curve FWHMs. As the Sc percentage increases, the surface changes from granular to atomic step flows (sample C8) with a reduction in the pit density to 2.0 × 10^8^ cm^−2^. However, at higher Sc-to-Al atomic flux ratios of 80%, the RHEED patterns become spotty, with giant hillocks of 300–400 nm in diameter formed on the surface. In Figure 5d, dual spots on both sides of the primary streaks indicate reflections from the (111) and (002) planes of rock-salt-phase Sc*_x_*Al_1−*x*_N [20,42]. EDX mappings in Figure 6a–c validate the homogeneous distribution of Sc and Al in sample C8. Figure 6d–g reveal that hillocks in samples C9 are Al-agglomerated, Sc-deficient microstructures, which potentially correspond to the metallic Al_3_Sc droplets observed via XRD (002) 2θ-ω scan [Figure 3b]. The dual spots of rock salt Sc*_x_*Al_1−*x*_N in RHEED [Figure 5d] are likely high-Sc-content alloys with XRD (002) 2θ-ω scan peak at ~34.5°obscured by the GaN peak [42]. Negligible desorption of Sc and Al adatoms is expected at T_sub_ = 680 °C. While the Sc composition in the wurtzite phase is almost identical for samples C8 and C9, a further increase in the Sc-to-Al atomic flux ratio from 60% (sample C8) to 80% (sample C9) led to Sc-deficient metallic Al_3_Sc and high-Sc-content rock salt Sc*_x_*Al_1−*x*_N.

**Figure 7:**
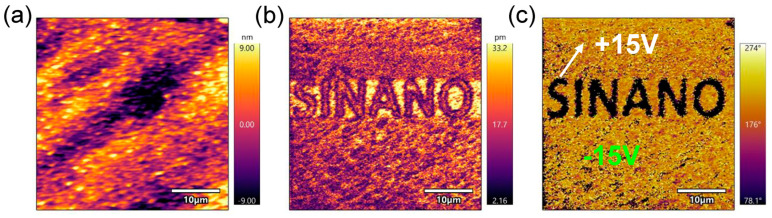
Piezoelectric force microscopy (PFM) analysis of the Sc_0.37_Al_0.63_N thin film grown on GaN/sapphire (samples C8): (**a**) surface morphology; (**b**) out-of-plane piezoelectric amplitude; and (**c**) phase diagrams under an external voltage of ±15 V versus the GaN buffer layer. The scan area is 40 μm × 40 μm, and the RMS is 4.41 nm.

In literature, a transition regime of mixed Sc*_x_*Al_1−*x*_N phases was universally observed. S. satoh et al. reported the transition from the wurtzite phase to the wurtzite-rock salt two-phase mixture at *x* = 0.30–0.35, and from the two-phase mixture to the rock salt phase at *x* = 0.38–0.43 [43]. Hoglund et al. reported that phase transition from a mixture of two phases of rock salt and wurtzite to a pure-rock-salt phase occurs at *x* = 0.40–0.48, depending on the seed layer [21,22]. In addition, Hoglund et al. reported phase separation of Sc*_x_*Al_1−*x*_N for *x* > 0.23 with the emergence of a rock salt ScN(111) peak in (002) 2θ-ω scans [21]. Herein, we identified the boundary of the stable wurtzite phase at *x* = 0.37–0.39, and in proximity to the boundary, we attained pure-wurtzite-phase smooth Sc_0.37_Al_0.63_N thin films based on XRD, RHEED, and EDX analysis. The piezoelectric response of the Sc_0.37_Al_0.63_N thin film (sample C8) was confirmed via PFM experiments. The surface morphology, out-of-plane piezoelectric amplitude, and phase diagrams in Figure 7 confirm that an external electric field of 1.5 MV/cm has selectively flipped the polarity by 180° with clear antiparallel domain boundaries. The piezoelectric amplitude at domain boundaries is at least one order of magnitude lower than that within each domain, which is a representative aspect of ferroelectricity due to the cancellation of polarization from opposite domains on either side.

Two different types of phase separation with degraded surface morphology have been identified in Sc*_x_*Al_1−*x*_N films grown under N-rich conditions. For the first scenario, a relatively high III/N atomic flux ratio of 0.89 with a fixed Sc-to-Al flux ratio causes Sc-agglomerated hillocks, whereas the target Sc content of *x* = 0.18 is much lower compared to the generally reported phase transition boundary of *x* ~ 0.30–0.43 [43]. Cubic-phase intermetallic Al_3_Sc was identified in sample C3 via XRD, which has higher Sc compared to the target Sc content of *x* = 0.18 in the wurtzite phase, and this is consistent with the observation of high-Sc-content, Al-deficient hillocks. Therefore, a sufficiently high over-flux of active N is mandatory to suppress Sc adatom migration and to facilitate the homogeneous incorporation of Sc into the wurtzite lattice. Boosting Sc adatom mobility with an increased III/N ratio, however, could drive the nucleation of thermodynamically preferential cubic-phase intermetallic Al_3_Sc and the rock salt phase of ScN, leading to high-Sc-content hillocks. For the second scenario with a fixed III/N ratio of 0.78, a high Sc-to-Al atomic flux ratio of 80% causes high-Sc-content rock-salt-phase Sc*_x_*Al_1−*x*_N, as evidenced by RHEED and intermetallic Al_3_Sc hillocks identified via XRD, which has a significantly higher Al percentage compared to the measured Al content of (1 − *x*) = 0.61 in the wurtzite phase. The high-Al-content, Sc-deficient hillocks observed via EDX indicates that the primary elemental inhomogeneity originates from Al_3_Sc, whereas the high-Sc-content rock-salt-phase Sc*_x_*Al_1−*x*_N could be uniformly embedded into the primary wurtzite phase thin film. The non-linear composition dependence of the bond angle and bond length at the phase transition boundary causes structural instability of the Sc*_x_*Al_1−*x*_N wurtzite lattice [37], which explains why the wurtzite lattice is “saturated” with the incorporation of 39% percent of Sc. Excessive Sc adatoms drive the formation of thermodynamically stable rock salt ScN and metallic Al_3_Sc. Therefore, the surface kinetics of Sc adatoms plays a critical role in the phase degradation of Sc*_x_*Al_1−*x*_N.

## 4. Conclusions

Wurtzite-phase Sc*_x_*Al_1−*x*_N (0.16 ≤ *x* ≤ 0.37) thin films were grown by precisely tuning the atomic fluxes of Sc, Al, and active N species. We identified two types of phase separation closely related to MBE growth kinetics. At the optimal III/N ratio of 0.78, increasing the Sc-to-Al flux ratio to >60% led to cubic-phase intermetallic Al_3_Sc and high-Sc-content rock-salt-phase Sc*_x_*Al_1−*x*_N, indicating a phase transition boundary at *x* = 0.37–0.39. EDX mappings proved the presence of high-Al-content hillocks across the sample surface. We hypothesized that structural instability of Sc*_x_*Al_1−*x*_N at the phase transition boundary promotes the surface accumulation of Sc adatoms, eventually forming undesirable phases. For the growth of Sc_0.18_Al_0.82_N, a relatively high III/N atomic flux ratio of 0.89 led to high-Sc-content hillocks, potentially due to enhanced Sc adatom migration with the increase in the III/N ratio. Our experiments have provided insights to the MBE growth diagram of wurtzite Sc*_x_*Al_1−*x*_N thin films.

We attained single crystalline wurtzite Sc_0.37_Al_0.63_N thin films with a surface roughness of 1.70 nm. The Sc composition of *x* = 0.37 was confirmed via XRD and XPS analysis, and the phase purity was verified via RHEED, XRD, and EDX. The XRD (002) and (102) ω rocking curve FWHMs of the Sc_0.37_Al_0.63_N thin film reached 2156 arcsec and 209 arcsec, respectively, superior to state-of-art high-Sc-content Sc*_x_*Al_1−*x*_N films [11,20]. PFM measurement proved that the polarization of the probed regions in the Sc_0.37_Al_0.63_N thin film can be switched 180° by an external electric field, manifesting the characteristic of ferroelectricity. Such single crystalline wurtzite Sc_0.37_Al_0.63_N thin films promise potential applications in the sub-6GHz resonant filter and high-density ferroelectric memory arrays.

## Data Availability

The data that supports the findings of this study is available within the article and its Supplementary Material. Additional data is available from the corresponding authors upon reasonable request.

## Supplementary Materials

The following supporting information can be downloaded at https://www.mdpi.com/article/10.3390/nano14171459/s1, See the supplementary materials for details on the EDX experiment and XRD (002) 2θ-ω scans of Sc_0.18_Al_0.82_N films grown on Si(111) substrate. References [11,14,20,25,44] is cited in Supplementary Materials.

## References

[1] Tasnádi F., Alling B., Höglund C., Wingqvist G., Birch J., Hultman L., Abrikosov I.A. (2010). Origin of the Anomalous Piezoelectric Response in Wurtzite Sc*_x_*Al_(1−*x*)_N Alloys. Phys. Rev. Lett..

[2] Akiyama M., Kano K., Teshigahara A. (2009). Influence of growth temperature and scandium concentration on piezoelectric response of scandium aluminum nitride alloy thin films. Appl. Phys. Lett..

[3] Akiyama M., Kamohara T., Kano K., Teshigahara A., Takeuchi Y., Kawahara N. (2009). Enhancement of Piezoelectric Response in Scandium Aluminum Nitride Alloy Thin Films Prepared by Dual Reactive Cosputtering. Adv. Mater..

[4] Yanagitani T., Suzuki M. (2014). Electromechanical coupling and gigahertz elastic properties of ScAlN films near phase boundary. Appl. Phys. Lett..

[5] Farrer N., Bellaiche L. (2002). Properties of hexagonal ScN versus wurtzite GaN and InN. Phys. Rev. B.

[6] Ambacher O., Christian B., Feil N., Urban D.F., Elsässer C., Prescher M., Kirste L. (2021). Wurtzite ScAlN, InAlN, and GaAlN crystals, a comparison of structural, elastic, dielectric, and piezoelectric properties. J. Appl. Phys..

[7] Fichtner S., Wolff N., Lofink F., Kienle L., Wagner B. (2019). AlScN: A III-V semiconductor based ferroelectric. J. Appl. Phys..

[8] Wang D., Wang P., Mondal S., Mohanty S., Ma T., Ahmadi E., Mi Z. (2022). An Epitaxial Ferroelectric ScAlN/GaN Heterostructure Memory. Adv. Electron. Mater..

[9] Yazawa K., Drury D., Zakutayev A., Brennecka G.L. (2021). Reduced coercive field in epitaxial thin film of ferroelectric wurtzite Al_0.7_Sc_0.3_N. Appl. Phys. Lett..

[10] Casamento J., Nguyen T.-S., Cho Y., Savant C., Vasen T., Afroz S., Hannan D., Xing H., Jena D. (2022). Transport properties of polarization-induced 2D electron gases in epitaxial AlScN/GaN heterojunctions. Appl. Phys. Lett..

[11] Wang P., Wang D., Wang B., Mohanty S., Diez S., Wu Y., Sun Y., Ahmadi E., Mi Z. (2021). N-polar ScAlN and HEMTs grown by molecular beam epitaxy. Appl. Phys. Lett..

[12] Frei K., Trejo-Hernández R., Schütt S., Kirste L., Prescher M., Aidam R., Müller S., Waltereit P., Ambacher O., Fiederle M. (2019). Investigation of growth parameters for ScAlN-barrier HEMT structures by plasma-assisted MBE. Jpn. J. Appl. Phys..

[13] Hardy M.T., Downey B.P., Nepal N., Storm D.F., Katzer D.S., Meyer D.J. (2017). Epitaxial ScAlN grown by molecular beam epitaxy on GaN and SiC substrates. Appl. Phys. Lett..

[14] Hardy M.T., Jin E.N., Nepal N., Katzer D.S., Downey B.P., Gokhale V.J., Storm D.F., Meyer D.J. (2020). Control of phase purity in high scandium fraction heteroepitaxial ScAlN grown by molecular beam epitaxy. Appl. Phys. Express.

[15] Mertin S., Heinz B., Rattunde O., Christmann G., Dubois M.-A., Nicolay S., Muralt P. (2018). Piezoelectric and structural properties of c-axis textured aluminium scandium nitride thin films up to high scandium content. Surf. Coat. Technol..

[16] Knisely K., Douglas E., Mudrick J., Rodriguez M., Kotula P. (2019). Thickness dependence of Al_0.88_Sc_0.12_N thin films grown on silicon. Thin Solid Films.

[17] Park M., Wang J., Dargis R., Clark A., Ansari A. Super high-frequency scandium aluminum nitride crystalline film bulk acoustic resonators. Proceedings of the 2019 IEEE International Ultrasonics Symposium (IUS).

[18] Sandu C.S., Parsapour F., Mertin S., Pashchenko V., Matloub R., LaGrange T., Heinz B., Muralt P. (2019). Abnormal Grain Growth in AlScN Thin Films Induced by Complexion Formation at Crystallite Interfaces. Phys. Status Solidi A.

[19] Mishin S., Oshmyansky Y. Optimizing high concentration Scandium Aluminum Nitride films. Proceedings of the 2023 IEEE International Ultrasonics Symposium (IUS).

[20] Hardy M.T., Lang A.C., Jin E.N., Nepal N., Downey B.P., Gokhale V.J., Scott Katzer D., Wheeler V.D. (2023). Nucleation control of high crystal quality heteroepitaxial Sc_0.4_Al_0.6_N grown by molecular beam epitaxy. J. Appl. Phys..

[21] Höglund C., Birch J., Alling B., Bareño J., Czigány Z., Persson P.O.Å., Wingqvist G., Zukauskaite A., Hultman L. (2010). Wurtzite structure Sc*_x_*Al_(1−*x*)_N solid solution films grown by reactive magnetron sputter epitaxy: Structural characterization and first-principles calculations. J. Appl. Phys..

[22] Höglund C., Bareño J., Birch J., Alling B., Czigány Z., Hultman L. (2009). Cubic Sc*_x_*Al_(1−*x*)_N solid solution thin films deposited by reactive magnetron sputter epitaxy onto ScN(111). J. Appl. Phys..

[23] Hirata K., Shobu K., Yamada H., Uehara M., Anggraini S., Akiyama M. (2020). Thermodynamic assessment of the Al–Sc–N ternary system and phase-separated region of the strained wurtzite phase. J. Eur. Ceram. Soc..

[24] Gund V., Davaji B., Lee H., Casamento J., Xing H.G., Jena D., Lal A. Towards Realizing the Low-Coercive Field Operation of Sputtered Ferroelectric Sc*_x_*Al_(1-*x*)_N. Proceedings of the 2021 21st International Conference on Solid-State Sensors, Actuators and Microsystems (Transducers).

[25] Engel Z., Motoki K., Matthews C.M., Doolittle W.A. (2022). Overcoming metal-rich surface chemistry limitations of ScAlN for high electrical performance heterostructures. J. Appl. Phys..

[26] Wang P., Wang B., Laleyan D.A., Pandey A., Wu Y., Sun Y., Liu X., Deng Z., Kioupakis E., Mi Z. (2021). Oxygen defect dominated photoluminescence emission of Sc*_x_*Al_(1−*x*)_N grown by molecular beam epitaxy. Appl. Phys. Lett..

[27] Casamento J., Chang C.S., Shao Y.-T., Wright J., Muller D.A., Xing H., Jena D. (2020). Structural and piezoelectric properties of ultra-thin Sc*_x_*Al_(1−*x*)_N films grown on GaN by molecular beam epitaxy. Appl. Phys. Lett..

[28] Hunter D.A., Lavery S.P., Edwards P.R., Martin R.W. (2022). Assessing the Impact of Secondary Fluorescence on X-Ray Microanalysis Results from Semiconductor Thin Films. Microsc. Microanal..

[29] Bryan I., Bryan Z., Mita S., Rice A., Tweedie J., Collazo R., Sitar Z. (2016). Surface kinetics in AlN growth: A universal model for the control of surface morphology in III-nitrides. J. Cryst. Growth.

[30] Heying B., Smorchkova I., Poblenz C., Elsass C., Fini P., Den Baars S., Mishra U., Speck J.S. (2000). Optimization of the surface morphologies and electron mobilities in GaN grown by plasma-assisted molecular beam epitaxy. Appl. Phys. Lett..

[31] Heying B., Averbeck R., Chen L.F., Haus E., Riechert H., Speck J.S. (2000). Control of GaN surface morphologies using plasma-assisted molecular beam epitaxy. J. Appl. Phys..

[32] Österlund E., Ross G., Caro M.A., Paulasto-Kröckel M., Hollmann A., Klaus M., Meixner M., Genzel C., Koppinen P., Pensala T. (2021). Stability and residual stresses of sputtered wurtzite AlScN thin films. Phys. Rev. Mater..

[33] Ambacher O., Mihalic S., Yassine M., Yassine A., Afshar N., Christian B. (2023). Review: Structural, elastic, and thermodynamic properties of cubic and hexagonal Sc*_x_*Al_(1−*x*)_N crystals. J. Appl. Phys..

[34] King-Smith R.D., Vanderbilt D. (1993). Theory of polarization of crystalline solids. Phys. Rev. B.

[35] Zoroddu A., Bernardini F., Ruggerone P., Fiorentini V. (2001). First-principles prediction of structure, energetics, formation enthalpy, elastic constants, polarization, and piezoelectric constants of AlN, GaN, and InN: Comparison of local and gradient-corrected density-functional theory. Phys. Rev. B.

[36] Fan S., Liu R., Huang Y., Liu J., Zhan X., Sun X., Feng M., Yin Y., Sun Q., Yang H. (2022). Observation of threading dislocations and misfit dislocation half-loops in GaN/AlGaN heterostructures grown on Si using electron channeling contrast imaging. J. Appl. Phys..

[37] Deng R., Evans S.R., Gall D. (2013). Bandgap in Al_(1−*x*)_Sc*_x_*N. Appl. Phys. Lett..

[38] Zywitzki O., Modes T., Barth S., Bartzsch H., Frach P. (2017). Effect of scandium content on structure and piezoelectric properties of AlScN films deposited by reactive pulse magnetron sputtering. Surf. Coat. Technol..

[39] Mayrhofer P.M., Eisenmenger-Sittner C., Stöger-Pollach M., Euchner H., Bittner A., Schmid U. (2014). The impact of argon admixture on the c-axis oriented growth of direct current magnetron sputtered ScxAl(1−x)N thin films. J. Appl. Phys..

[40] Zhang Z., Zhang L., Wu Z., Gao M., Lou L. (2023). Characterization of AlN thin film piezoelectric coefficient d_31_ using Piezoelectric Micromachined Ultrasonic Transducers (PMUTs). J. Phys. Conf. Ser..

[41] Wurm C., Ahmadi E., Wu F., Hatui N., Keller S., Speck J., Mishra U. (2020). Growth of high-quality N-polar GaN on bulk GaN by plasma-assisted molecular beam epitaxy. Solid State Commun..

[42] Casamento J., Wright J., Chaudhuri R., Xing H., Jena D. (2019). Molecular beam epitaxial growth of scandium nitride on hexagonal SiC, GaN, and AlN. Appl. Phys. Lett..

[43] Satoh S., Ohtaka K., Shimatsu T., Tanaka S. (2022). Crystal structure deformation and phase transition of AlScN thin films in whole Sc concentration range. J. Appl. Phys..

[44] Wang D., Wang P., Mondal S., Liu J., Hu M., He M., Nam S., Peng W., Yang S., Wang D. (2023). Controlled ferroelectric switching in ultrawide bandgap AlN/ScAlN multilayers. Appl. Phys. Lett..

