# Supplementary material for: High-Quality Single Crystalline Sc0.37Al0.63N Thin Films Enabled by Precise Tuning of III/N Atomic Flux Ratio during Molecular Beam Epitaxy
Source: Nanomaterials (Basel). 2024 Sep 8;14(17):1459. doi: 10.3390/nano14171459 (PMC11396886; doi:10.3390/nano14171459)
Supplement: Supplementary file 1 [file nanomaterials-14-01459-s001.zip › nanomaterials-3163952-supplementary.pdf]

Article

High-Quality Single Crystalline $Sc_{0.37}Al_{0.63}N$ Thin Films Enabled by Precise Tuning of III/N Atomic Flux Ratio during Molecular Beam Epitaxy

Yuhao Yin [1,†], Rong Liu [1,†], Haiyang Zhao [1,†], Shizhao Fan [1,*], Jianming Zhang [2], Shun Li [2], Qian Sun [1,*] and Hui Yang [1]

[1] Key Laboratory of Semiconductor Display Materials and Chips, Suzhou Institute of Nano-Tech and Nano-Bionics, Chinese Academy of Sciences, Suzhou 215123, China

[2] Institute of Quantum and Sustainable Technology (IQST), School of Chemistry and Chemical Engineering, Jiangsu University, Zhenjiang 212013, China

* Correspondence: szfan2020@sinano.ac.cn (S.F.); qsun2011@sinano.ac.cn (Q.S.)

† These authors contributed equally to this work.

S1. EDX experiment on $Sc_xAl_{1-x}N$ epilayers.

Figure.S1 (a) The EDX spectra for sample C3 ($Sc_{0.18}Al_{0.82}N$ grown on Si, III/N = 0.89) and (b) sample C9 ($Sc_{0.39}Al_{0.61}N$ grown on GaN/sapphire, III/N = 0.78) taken at 5 keV (black) and at 10 keV (red). The inset shows a magnified view of the Sc-Kα peak in the range of 3.5—5.0 keV.

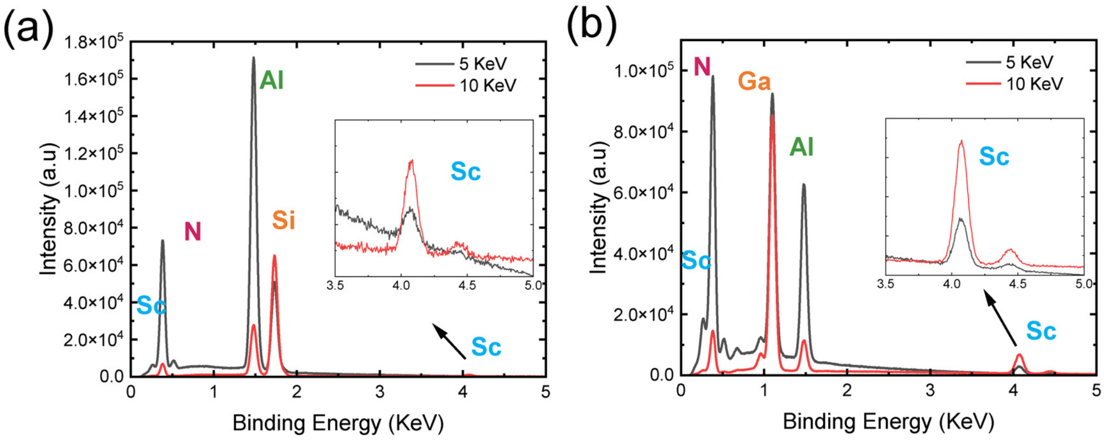

We surveyed multiple spots by EDX mappings and line scans across the 2-inch wafer of sample C2 ($Sc_{0.18}Al_{0.82}N$ grown on Si, III/N = 0.78), with the EDX mappings and line scans of three representative spots is shown in Fig. SM2. Please note that our nitrogen plasma cell sustains a uniform flux of active N species within a diameter of ~ 35 mm at the substrate growth position, which is the focused region in our study. The external ring region of the 2-inch wafer (<10 mm in width from the wafer edge)

corresponds to N-deficient growth condition. Our EDX analysis revealed uniform distribution of Sc and Al. In contrast, the EDX line scans for sample C3 ($Sc_{0.18}Al_{0.82}N$ grown on Si, III/N = 0.89) demonstrates an uneven distribution of Sc and Al in Fig. 2(g). This discrepancy is primarily attributed to the presence of surface hillocks. The hillocks exhibit higher Sc content and lower Al content compared to the surrounding regions. The comparison between the smooth sample C2 and the hillock-covered sample C3 highlights the effectiveness of the optimized III/N ratio of 0.78 in achieving a uniform film composition.

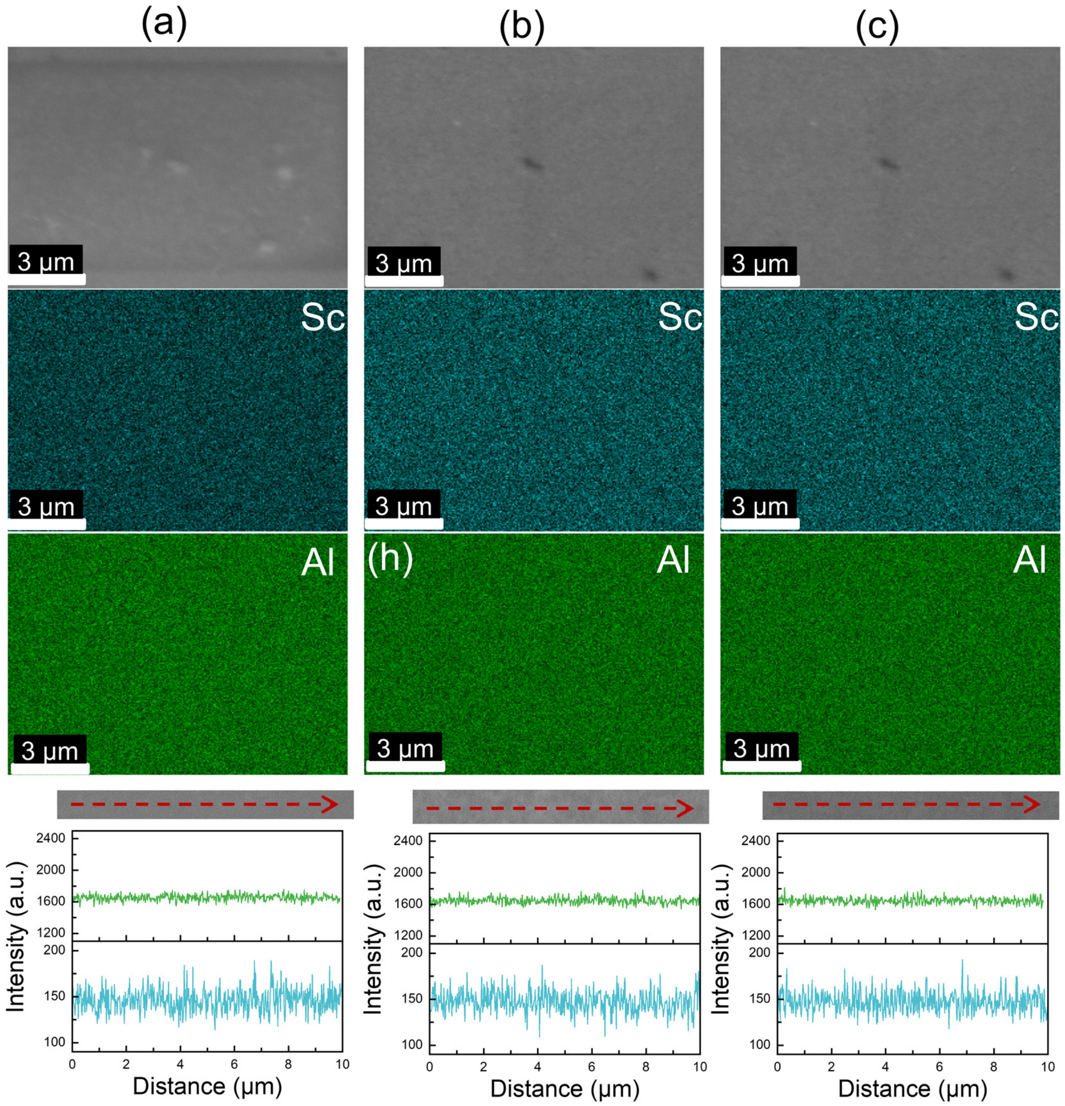

Figure S2. EDX analysis of sample C2 ($Sc_{0.18}Al_{0.82}N$ grown on Si, III/N = 0.78) (a) at the center of the wafer, (b) at 5 mm from the center, and (c) at 10 mm from the center to show uniform distribution of Sc and Al. The electron beam energy is 10 keV for all EDX mappings and the line scan of Al (green). The beam energy is 5 keV for the line scan of Sc (cyan).

S2. XRD (002) 2θ-ω scans of $Sc_{0.18}Al_{0.82}N$ films grown on Si(111) substrate.

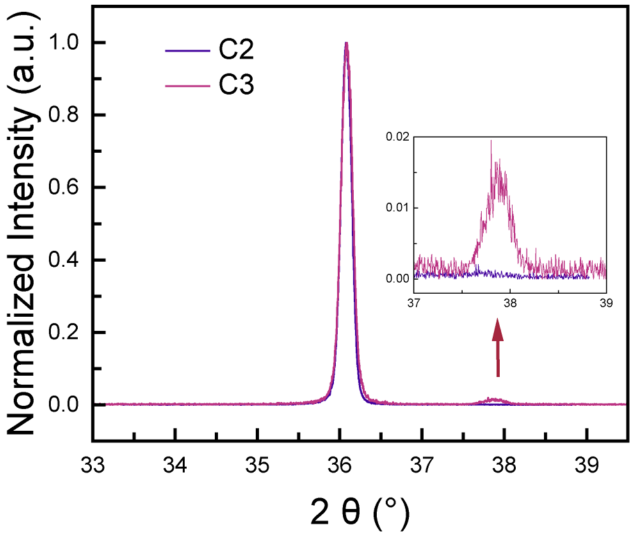

Figure S3. Comparison of XRD (002) 2θ-ω scans of $Sc_{0.18}Al_{0.82}N$ films. The blue and orange curves represent samples C2 and C3, respectively. The peak at 37.9° due to the metallic phase Al_3Sc [1] is observed in sample C3 (III/N = 0.89) but not in sample C2 (III/N=0.78), validating phase separation in sample C3.

S3. Crystal quality comparison of high-Sc-content $Sc_xAl_{1-x}N$ epilayers.

Table S1. Comparison of (002) and (102) double crystal XRD ω rocking curve FWHMs of high-Sc-content $Sc_xAl_{1-x}N$ epilayers in literature.

x of $Sc_xAl_{1-x}N$	(002) FWHM (arcsec)	(102) FWHM (arcsec)	Reference
0.32	1840	—	M. Hardy *et al.*[2]
0.39	3190	—	M. Hardy *et al.*[3]
0.30	2200	—	P. Wang *et al.*[4]
0.38	2800	—	
0.25	2088	4320	D. Wang *et al.*[5]
0.27	911	256	This work
0.37	2156	209	

REFERENCES

1. Engel, Z., et al., *Overcoming metal-rich surface chemistry limitations of ScAlN for high electrical performance heterostructures.* Journal of Applied Physics, 2022. **132**(18).
2. Hardy, M.T., et al., *Control of phase purity in high scandium fraction heteroepitaxial ScAlN grown by molecular beam epitaxy.* Applied Physics Express, 2020. **13**(6): p. 065509.
3. Hardy, M.T., et al., *Nucleation control of high crystal quality heteroepitaxial Sc0.4Al0.6N grown

by molecular beam epitaxy. Journal of Applied Physics, 2023. **134**(10).
4. Wang, P., et al., *N-polar ScAlN and HEMTs grown by molecular beam epitaxy.* Applied Physics Letters, 2021. **119**(8).
5. Wang, D., et al., *Controlled ferroelectric switching in ultrawide bandgap AlN/ScAlN multilayers.* Applied Physics Letters, 2023. **123**(10).
